# The psychosis analysis in real-world on a cohort of large-scale patients with schizophrenia

**DOI:** 10.1186/s12911-020-1125-0

**Published:** 2020-07-09

**Authors:** Wenyan Tan, Haicheng Lin, Baoxin Lei, Aihua Ou, Zehui He, Ning Yang, Fujun Jia, Heng Weng, Tianyong Hao

**Affiliations:** 1 Guangdong Mental Health Center, Guangdong Provincial People’s Hospital, Guangdong Academy of Medical Science, Guangzhou, China; 2 School of Computer Science, South China Normal University, Guangzhou, China; 3 Department of Big Data Research of Medicine, The Second Affiliated Hospital of Guangzhou University of Chinese Medicine, Guangzhou, China; 4 The Affiliated Brain Hospital of Guangzhou Medical University (Guangzhou Huiai Hospital), Guangzhou, China

**Keywords:** Schizophrenia, Epidemiology research, Quantitative analysis

## Abstract

**Background:**

With China experiencing unprecedented economic development and social change over the past three decades, Chinese policy makers and health care professionals have come to view mental health as an important outcome to monitor. Our study conducted an epidemiological study of psychosis in Guangdong province, with 20 million real-world follow-up records in the last decade.

**Methods:**

Data was collected from Guangdong mental health information platform from 2010 to 2019, which had standardized disease registration and follow-up management for nearly 600,000 patients with six categories of mental diseases and 400,000 patients with schizophrenia. We conducted clinical staging for the disease course of the patients and divided the data with various factors into different stages of disease. Quantitative analysis was utilized to investigate the high relevant indicators to the disease. The results were projected on geography map for regional distribution analysis.

**Results:**

The majority cases of mental disease incidence were between the age of 15 and 29, while the peak age for both male and female was between 20 to 24 years old. The disease course with the largest number of patients’ cases was between 5 to 10 years. The therapeutic effect of patients gradually decreased with the development of disease course, while the risk increased with the disease course. The analysis of influencing factors showed that poor economic conditions incurred higher risk scores, and good medication adherence was effective in improving treatment outcomes. In addition, receiving good education contributed to the reduction of the risk of schizophrenia and the improvement of the efficiency of early treatment. Through the analysis of regional distribution of schizophrenia disease, developed economic conditions and favorable resource conditions could promote the reduction of disease risk, while in economically backward regions, it often accompanied with lower therapeutic effect and higher disease risk.

**Conclusions:**

Certain demographic factors had a relatively prominent impact on the therapeutic effect and risk of schizophrenia, such as high-quality medication adherence. Therapeutic effect and risk were highly correlated. Backward economic conditions often associated with poor efficacy and higher risk assessment, and the developed economy and better medical resource are beneficial for the treatment of psychotic.

## Background

Schizophrenia is a chronic disorder characterized with frequent relapses that have severe consequences for patients’ quality of life, including occupational and psychosocial functions, as well as economic burden for the patients and their families [1]. Among the patients with schizophrenia, the first onset of mental disorders usually occurs in childhood or adolescence [2]. In addition, the mortality of patients with the disease is relatively high than normal people [3, 4], and this mortality gap is continually increasing in recent decades [5]. Although the burden of schizophrenia is increasing globally, it is still among incurable diseases even its causes remain unknown. Given the severity, consequences and unknown etiology, more attention needs to be paid to the study of potential risk factors of the disease and to contribute to prevention of the condition [6].

In recent years, there is an increasing number of epidemiology studies on schizophrenia [7]. The practical purpose of epidemiology is discovering relations which offer possibilities for disease prevention [8], and the epidemiology counts health-related phenomenon to map the frequency and distribution of disease across time and space. This helps answer the questions related to which risk factors are linked to mental disorders and which treatments and services can influence the course of mental disorders [9]. McGrath et al. (2008) [10] concluded that the epidemiology of schizophrenia is characterized by prominent variability and gradients that could help guide future research. Hence, many epidemiology studies have been concerned with the impact of the environment on the development of schizophrenia. The marked variation in incidence of schizophrenia is existed in various aspects [11], and several socio-environmental factors have been proposed to explain the prominent variation, such as men are at higher risk of all psychotic disorders than women [12] and the rates increase for ethnic minority groups, as well as with lower socioeconomic status and in urban and deprived neighborhoods [13, 14]. In view of these factors on the impact of schizophrenia, it is essential to capture at least some of these gradients and the clues that have been generated by epidemiology [15].

In China, schizophrenia is the most common diagnosis among hospitalized psychiatric patients [16], and it has the highest age-standardized prevalence of schizophrenia compared with other countries [17]. The early two large-scale epidemiological surveys on mental disorders in China were conducted in 1982 and 1993, providing valuable foundations and guidance for next research. With the fast development of China’s economy and the accompanying changes in demographic structure, education, social status and other aspects, the prevalence of most mental disorders, however, is showing an increasing trend [18]. Along with the continuous improvement of population quality, the Ministry of Health and local government, as well as the society, have realized the importance and consequence of mental disorder [19], and the China Mental Health Survey (CMHS) was launched in 2012, which is aimed to conduct the nationally representative community survey on mental disorders in China [20, 21]. A meta-analysis estimated the pooled prevalence of schizophrenia and suggested that location distribution affects the prevalence estimates significantly, with the prevalence for urban residents was higher than for rural [22]. Another study demonstrated that family economic status might also contribute to schizophrenia as a potential impact [10]. Except for national studies, another analysis conducted a series epidemiological studies in selected four provinces to explore the prevalence of different types of mental disorders in China [23]. These epidemiological studies investigated the influence factors and prevalence in China. Due to the large population base of China, a low prevalence still can cover an enormous number of people. Thus, it is urgently needed to take effort to deal with the huge unmet need for treatment of mental disorders [24].

Based on the previous epidemiological studies of risk factors for schizophrenia, we used statistical analysis to investigate the impact of relative factors on schizophrenia. We first conducted a preliminary analysis of age, course of disease and geographical distribution. In order to explore the influence of relevant factors on schizophrenia, we selected several valuable attributes based on the records of patients’ relevant information in the follow-up data to evaluate the effect of these attributes on the treatment effect and risk of schizophrenia. Finally, we explored the regional distribution characteristics of the disease as well as analyzed the related phenomena, which indicates several severely affected areas that we should pay much attention to. To our knowledge, this is the first and largest quantitative study on the epidemiology of mental disorders based on the large numbers of follow-up data in Guangdong province, which is the province with largest economy volume in the country.

The contribution of this work is as follows: (1) Collecting and preprocessing the largest study data containing approximately 20 million real-world follow-up records and nearly 400,000 patients with schizophrenia in Guangdong province. (2) Defining measures to analyze the treatment effect and risk, and analyzing the influence factors, as well as comparing their difference between groups. (3) Exploring the characteristics of the regional distribution of schizophrenia and comparing the differences of the treatment effect and risk in different regions, which provided a reference for the effective and reasonable allocation of medical resources and the optimization of disease prevention and control. (4) Discussing how these factors can be applied to clinical systems or medical institutions by analyzing the factors that influence schizophrenia for applying the optimized outcomes to prediction, diagnosis, or treatment.

## Methods

### Design

**Fig. 1:**
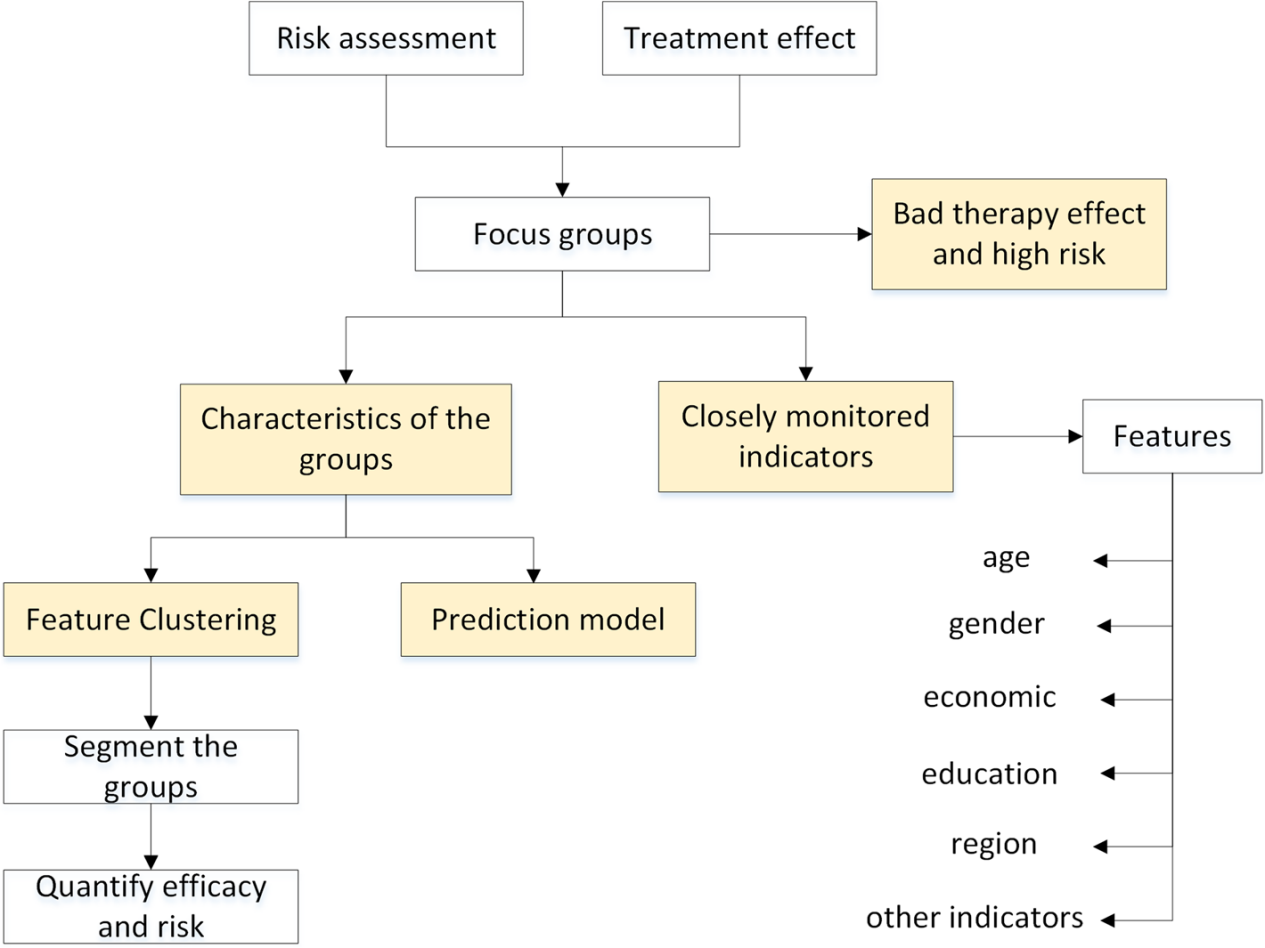
The overall framework of analysis

The overall analytical framework is shown in Fig. 1. From the risk and treatment effect existing in the follow-up data, we extracted the focus groups with negative therapy effect and high risk. Age, gender, blood glucose and other indicators were as the closely monitored indicators. The characters of group were used to design the prediction model and feature clustering, which was used to analyze the group and quantify treatment effect and risk. The quantified treatment effect and risk were used afterwards for estimating the influence of different factors in schizophrenia.

### Data preparation

The study population including 348,801 individuals with valid registered records who living in Guangdong province and were diagnosed with ICD code F20.* during the period from January 1, 2010 to May 30, 2019, of which 1755 were excluded due to missing or implausible data. The follow-up data was obtained from the Guangdong Mental Health center network medical System (GDMHS), which was initiated in 2000 and covered over 99% of schizophrenia patients in the province. Within the disease registration report system, patient profile, diagnosis and treatment information in hospitals were reported and recorded into the system via a computer network.

Validity of the data was confirmed by psychiatrist doctors and a chief physician in the hospital after data entry. Moreover, the Health Commission verified the data annually through sampling survey to ensure data reliability. Since the data from GDMHS did not contain individually identifiable information, and therefore individual informed consent was not required.

### Covariates

We conducted a descriptive analysis of the data based on age groups and disease course length. The evaluation models of therapeutic effect and risk were focusing on gender, economic status, medication adherence, education level, risk evaluation, region distribution, register type and age group. The data was also represented as points and density levels to project them geographically for presenting the location characteristics of patients. The location factor was taken into account in order to investigate the regional distribution of mental disorders.

### Evaluation metrics

The measures of main treatment outcome in this study were quantified treatment effect and risk. The calculation method of the efficiency rate is as Eq. (1).

1 $$ Efficiency=\frac{\left|\mathrm{C} ured\right|+\left| Improved\right|}{\left| Total\right|} $$

where *Cured* is the patients cured, *Improved* is the patient has improvement on disease, and *Total* is the total follow-up records of this research group.

During the evaluation of treatment risk, the assessment can be classified into six levels based on the differences of patients’ symptoms, as shown in Table 1.

**Table 1:** Classification of risk assessment levels

Level 0	None of the following behaviors are consistent with levels 1–5.
Level 1	Verbal threats and shout but no hitting behaviors.
Level 2	Hitting behavior is confined to the home and can be persuaded to stop.
Level 3	Having obvious hitting behaviors which regardless of the occasion and only aim at the property and items, cannot persuaded to stop.
Level 4	Persistent hitting behaviors which regardless of the occasion and target at property or people, cannot be persuaded to stop (including self-injury and suicide).
Level 5	Any act of violence against a person with a weapon, or an act of explosion, arson, etc., whether at home or in public.

### Statistical analysis

In this study, we adopted various statistical analysis methods to explore the influence of factors on the incidence of outcomes to reveal the treatment characteristics of real-world schizophrenia at different stages of disease course as well as relevant factors affecting the treatment effect and risk of schizophrenia.

To assess the therapeutic characteristics of schizophrenia in different populations and stages of the disease, we grouped patients according to different disease courses and calculated proportion of each factor in different disease stages. The influence of each factor on the indicators was divided into five different stages: 0–6 months, 7–12 months, 13–24 months, consolidation period (2–5 years), and maintenance period (over 5 years). In addition, we sorted and grouped different attributes for each factor, such as households was grouped as urban and rural areas, economic conditions was grouped as poverty and non-poverty, etc., to explore the intra-group differences in the influence of different factors.

In the descriptive analysis, we conducted a statistical analysis towards the data. We grouped patients by age and gender, to observe the number of patients in different age groups and whether there existed differences between different gender groups. In the meantime, the course of disease was grouped according to the follow-up time points, and analyzed count and variation trends of male and female cases in different disease courses respectively. Finally, the regional distribution of disease was depicted based on the follow-up sites of mental patients to analyze the regional characteristics of the incidences of schizophrenia.

During the quantitative analysis of treatment and risk, the patient treatment data, such as treatment development status including cure, improvement, no-change or aggravation, was recorded. In order to calculate efficiency and risk scores, we grouped the data by course of disease in year, and then summarized the follow-up records by year and patient ID. According to a list of evaluation metrics, the total effective rate and risk of each patient per year were calculated. After that, the mean (95% confidence interval) was used to calculate the total effective rate and risk per year. We used the time length of the disease as an independent variable to investigate the variation trend of different factors on the time axis of the disease course towards efficiency and risk, and to compare the differences between different attributes of the same factor. In order to further analyze the trend of schizophrenia among different factors, the Cox-Stuart method was used to test the significance of the risk and efficacy trend of each group to explore the changing trend of risk and efficacy of different attributes in each factor group with the course of disease. In risk assessment, if a patient appeared with multiple different evaluation levels, level 0 to 2 was represented as 0 point and level 3 to 5 was represented 1 point. Then the amount of times appeared a year is counted as the count of corresponding points.

In addition, we investigated the characteristics of the regional distribution of treatment effects and risks, which were conducive to the early detection of problems from geographical distribution. A hotspot map was utilized to represent the distributions of relevant indicators of patients. First, we exploited a gradient of color levels to map the patient population density in different areas. Then, multiple groups of efficient intervals were divided and the interval values were indicated by the depth of color, with the deeper color representing the higher efficient value. Efficient mappings were represented in node color on the map. In order to map the risk assessment, we used risk scores as a variable and plotted them as the sizes of nodes, with the larger nodes representing higher risk score in the region. Meanwhile, with the goal of analyzing the demographic characteristics of a region, the average age and length of disease course of the patients in the region were counted to be the labels of the nodes.

Analyses were stratified to examine the epidemiology of psychosis in Guangdong province from different perspectives, with exploring the characteristics of psychosis population, and the differences and evolution trends of schizophrenia in urban and rural, region, economic status, education status and other aspects.

## Results

From 10-year follow-up data of psychiatric patients, 348,801 individuals were screened, where 190,225 (54.5%) patients were male and 158,576 (45.5%) were female. Among the patients, 144,804 (41.6%) were registered in urban areas, while 203,373 (58.4%) were registered in rural areas. Meanwhile, 208,894 (59.9%) of the patients were recorded as poverty population and only 139,683 (40.1%) were non-poverty population. Young and middle-aged patients accounted for most of the follow-up population, among which 149,295 (42.8%) were young (18–44 years old) and 148,348 (42.5%) were middle-aged and elderly (45–65 years old) at all stages of the disease. Meanwhile, most of these follow-up patients only received primary education (224,330, 64.3%), followed by illiteracy & semi-illiteracy (55,915, 16.0%). Only 10,331 (3.3%) patients experienced higher education.

### Group analysis of disease course

The statistical information of social demographic characteristics was shown in Table 2. We first analyzed the internal characteristics of the follow-up data in groups. Comparing the distribution of various factors at different stages, the count of patients at different stages of the disease presented an increasing trend. The number of patients in the maintenance period was the most, while patients at the onset of 0–6 months was the least. According to the follow-up age, young people and middle-aged and elderly people were the main population with the disease, representing that the disease age was mainly distributed between 18 and 65 years old. In terms of geographical category, the population groups of patients in Guangdong province were mainly distributed in the economically developed areas, e.g., Guangzhou, Foshan and Shenzhen, as well as the densely populated areas, e.g., Chaoshan, and Meizhou. In addition, most schizophrenic patients had a low level of education, and the poverty rural areas having more patients may also represent a lower level of education, compared with the non-poor urban areas.

**Table 2:** The statistics of follow-up data in stages according to the disease course of patients

Disease course classification	Total	0 ~ 6 months	7 ~ 12 months	13 ~ 24 months	Consolidation	Maintenance	Sum (Ratio)
		# Cases (Ratio)	# Cases (Ratio)	# Cases (Ratio)	# Cases (Ratio)	# Cases (Ratio)	
Group summaries	348,801	2657 (0.76%)	3971 (1.14%)	13,260 (3.80%)	57,822 (16.58%)	271,091 (77.72%)	
Sex	348,801						
1-Male		1410 (0.40%)	2199 (0.60%)	7187 (2.10%)	31,484 (9.00%))	147,945 (42.40%)	190,225 (54.5%)
2-Female		1247 (0.40%)	1772 (0.50%)	6073 (1.70%)	26,338 (7.60%)	123,146 (35.30%)	158,576 (45.5%)
Age	348,737						
01.Child 0 ~ 6		–	–	–	7 (0.00%)	2 (0.00%)	9 (0.0%)
02.Youngster 7 ~ 12		5 (0.00%)	11 (0.00%)	10 (0.00%)	71 (0.00%)	94 (0.00%)	191 (0.1%)
03.Adolescent 13 ~ 18		108 (0.00%)	153 (0.00%)	391 (0.10%)	689 (0.20%)	494 (0.10%)	1835 (0.5%)
04.Youg people 18 ~ 44		1473 (0.40%)	2103 (0.60%)	7330 (2.10%)	32,207 (9.20%)	106,182 (30.40%)	149,295 (42.8%)
05.Middle-aged 45 ~ 65		800 (0.20%)	1253 (0.40%)	4098 (1.20%)	18,495 (5.30%)	123,702 (35.50%)	148,348 (42.5%)
06.Old age 65~		270 (0.10%)	450 (0.10%)	1429 (0.40%)	6338 (1.80%)	40,572 (11.60%)	49,059 (14.1%)
Registration	348,177						
1-Urabn		910 (0.30%)	1331 (0.40%)	5179 (1.50%)	22,484 (6.50%)	114,900 (33.00%)	144,804 (41.6%)
2-Rural		1732 (0.50%)	2618 (0.80%)	8030 (2.30%)	35,183 (10.10%)	155,810 (44.80%)	203,373 (58.4%)
Regions	348,801						
1-Guangzhou &Foshan&Shenzhen		310 (0.10%)	488 (0.10%)	1582 (0.50%)	6714 (1.90%)	44,447 (12.70%)	53,541 (15.4%)
2-Meizhou&Chaoshan		346 (0.10%)	666 (0.20%)	2170 (0.60%)	8542 (2.40%)	46,014 (13.20%)	57,738 (16.6%)
3-South coastal region		290 (0.10%)	411 (0.10%)	1435 (0.40%)	6336 (1.80%)	33,278 (9.50%)	41,750 (12.0%)
4-Xinfeng&Heyuan		103((0.00%))	149 (0.00%)	530 (0.20%)	2198 (0.60%)	11,208 (3.20%)	14,188 (4.1%)
5-Remote areas in northern Guangdong		157 (0.00%)	205 (0.10%)	763 (0.20%)	3321 (1.00%)	17,427 (5.00%)	21,873 (6.3%)
6-Northern Guangdong&Shaoguan		275 (0.10%)	361 (0.10%)	1058 (0.30%)	6246 (1.80%)	25,741 (7.40%)	33,681 (9.7%)
7-Mountainous area in western Guangdong		211 (0.10%)	297 (0.10%)	785 (0.20%)	4044 (1.20%)	18,116 (5.20%)	23,453 (6.7%)
8-West coast in Guangdong		12 (0.00%)	43 (0.00%)	115 (0.00%)	431 (0.10%)	549 (0.20%)	1150 (0.3%)
0-Other regions		953 (0.30%)	1351 (0.40%)	4822 (1.40%)	19,990 (5.70%)	74,311 (21.30%)	101,427 (29.1%)
Educational status	348,801						
Unknown		330 (0.10%)	493 (0.10%)	1698 (0.50%)	7347 (2.10%)	14,857 (4.30%)	24,725 (7.1%)
Primary education		1555 (0.40%)	2305 (0.70%)	7932 (2.30%)	34,686 (9.90%)	177,852 (51.00%)	224,330 (64.3%)
Higher education		160 (0.00%)	189 (0.10%)	551 (0.20%)	2156 (0.60%)	7275 (2.10%)	10,331 (3.0%)
Secondary school education		328 (0.10%)	429 (0.10%)	1368 (0.40%)	5580 (1.60%)	25,795 (7.40%)	33,500 (9.6%)
Illiteracy & semi-illiteracy		284 (0.10%)	555 (0.20%)	1711 (0.50%)	8053 (2.30%)	45,312 (13.00%)	55,915 (16.0%)
Economic status	348,577						
1-Poverty		1192 (0.30%)	2056 (0.60%)	6436 (1.80%)	32,069 (9.20%)	167,141 (47.90%)	208,894 (59.9%)
2-Non-poverty		1455 (0.40%)	1908 (0.50%)	6809 (2.00%)	25,706 (7.40%)	103,805 (29.80%)	139,683 (40.1%)

### Descriptive analysis

**Fig. 2:**
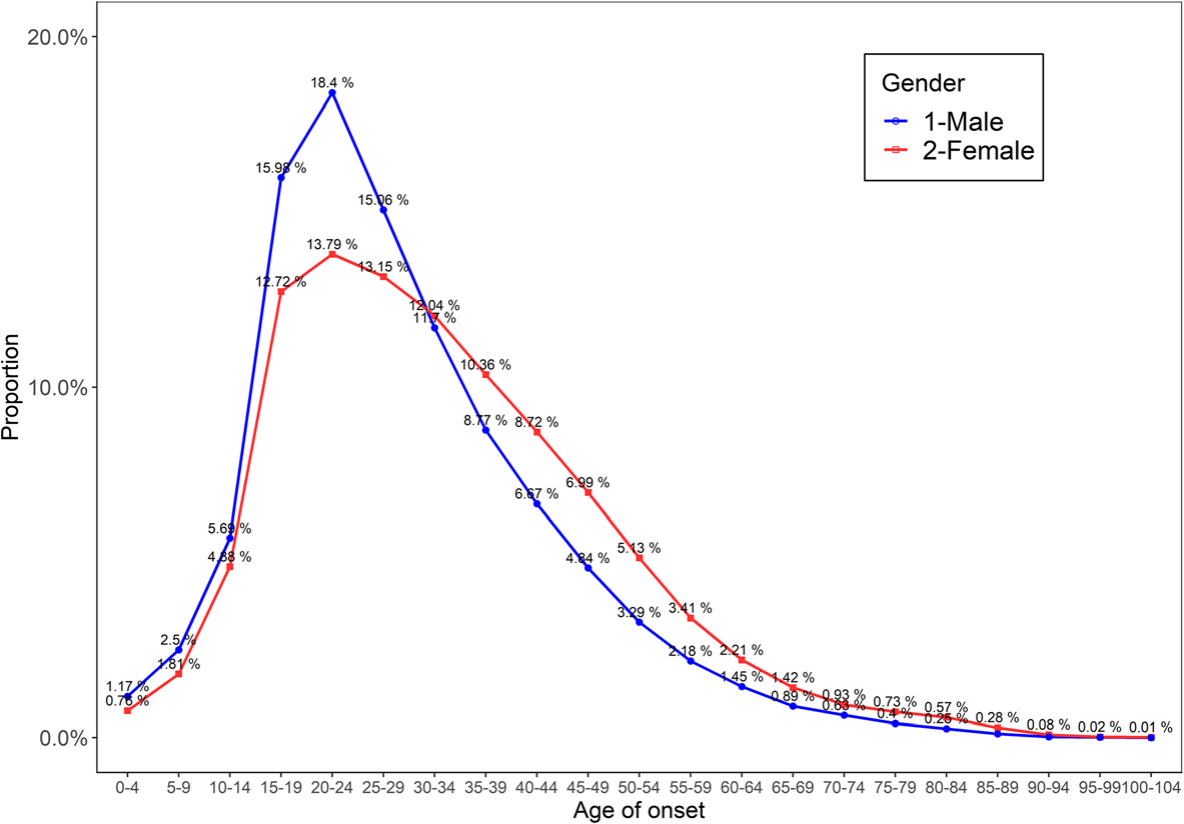
The proportion of patients with different onset ages. The two lines represent the distributions of male and female patients

**Fig. 3:**
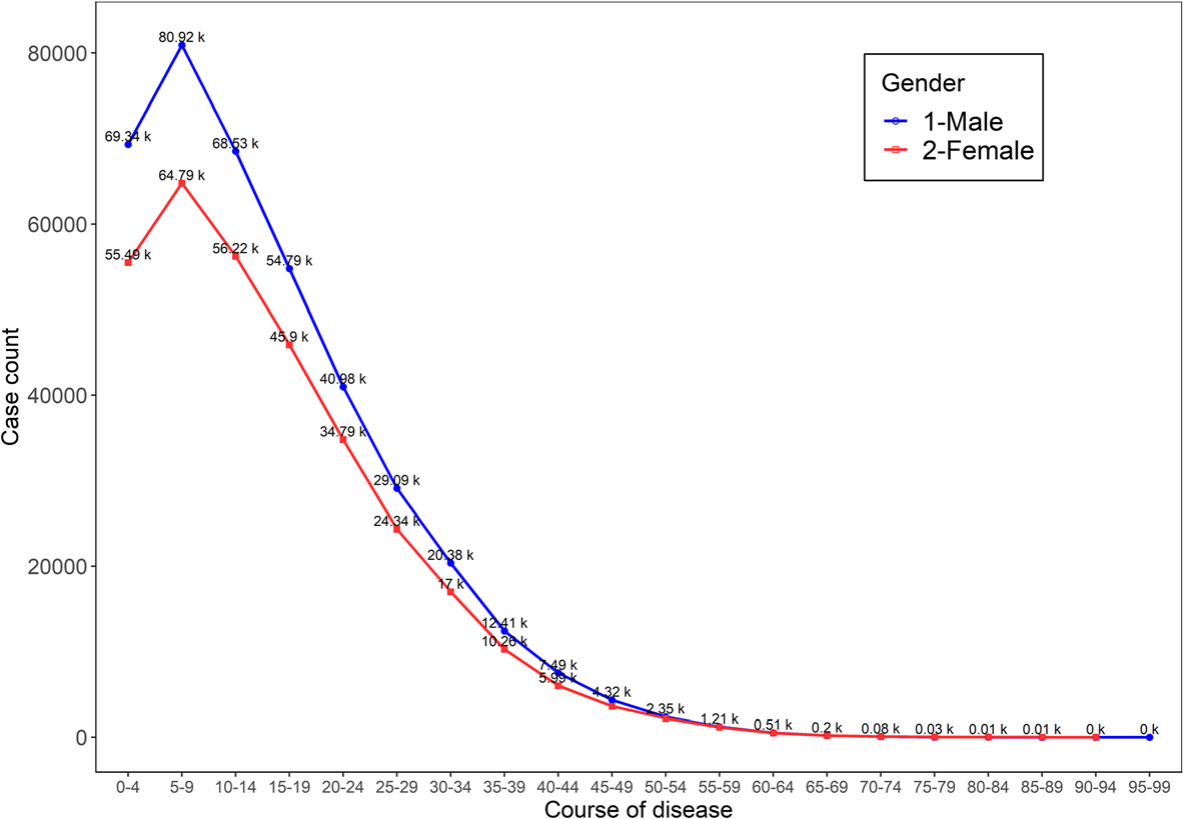
The distribution of course of disease in patients with schizophrenia by gender

Among the follow-up data, the number of cases at the age of initial onset (in units of 10) were grouped and summarized, and the distribution of onset age were shown in the Fig. 2. The highest incidence of schizophrenia in both men and women was aged between 20 and 24, and the count of patients between 15 to 29 years old was the majority. Also, there were more patients in men before the age of 30–34 years old than in women, and fewer patients after the age of 34. In view of such situation, it was appeared to be attributed to men may be related to work pressure and women may be related to menopause physiology. We also observed the variation in the number of patients with schizophrenia over course of disease (respectively observed in men and women). As shown in the Fig. 3, by dividing the course of disease into different intervals, most patients’ course of disease was concentrated between 5 and 10 years. The number of cases with course of disease increased from 0 to 10 years, and then presented a trend of decline. Meanwhile, the result demonstrated that men patients had more cases per course of disease than women.

### Quantitative analysis of efficacy and risk

We first computed the effectiveness and risk of each factor using a list of quantitative strategies aforementioned. According to the social demography of the data, the effects of various factors with the time intervals of disease course on the efficiency and risk were analyzed. Finally, we explored the efficiency and risk of trends of disease course in eight groups respectively.

**Fig. 4:**
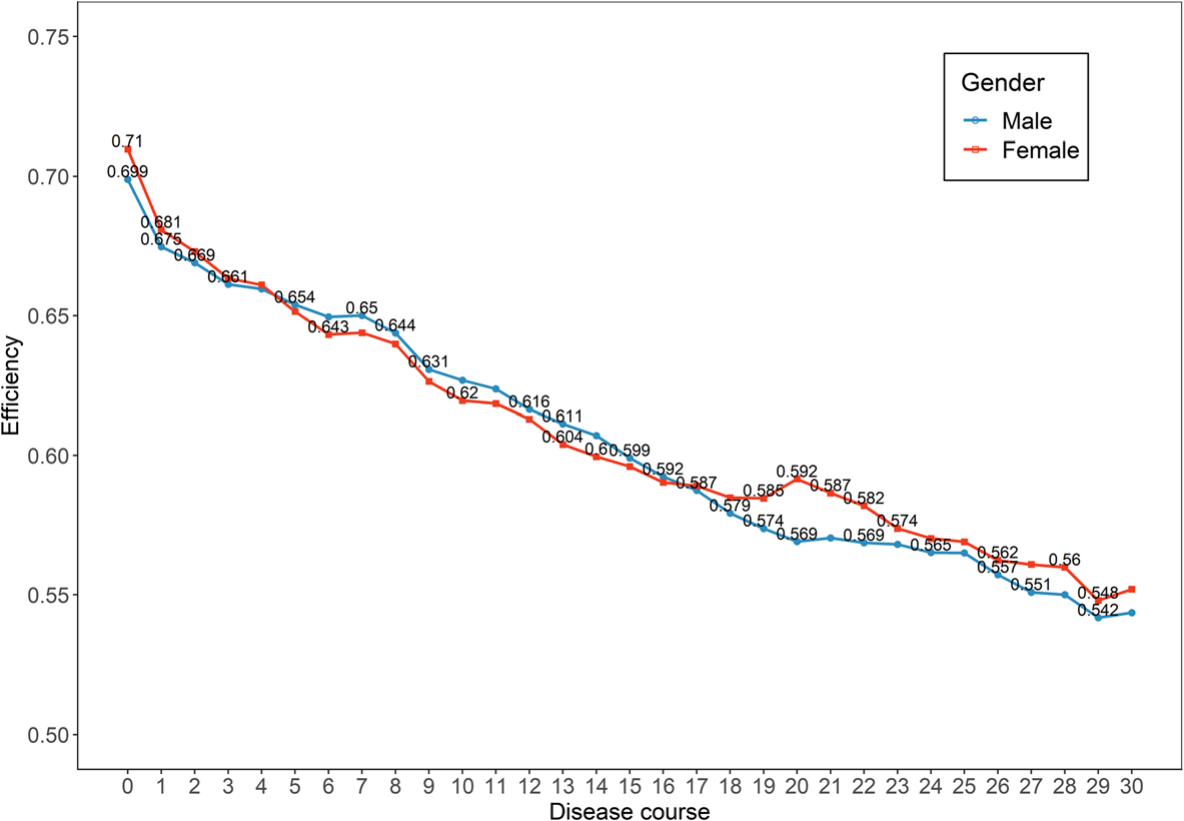
The change of effective rate with the course of disease by gender with decreasing trend (Male, *p* < 0.001; Female, *p* < 0.001)

**Fig. 5:**
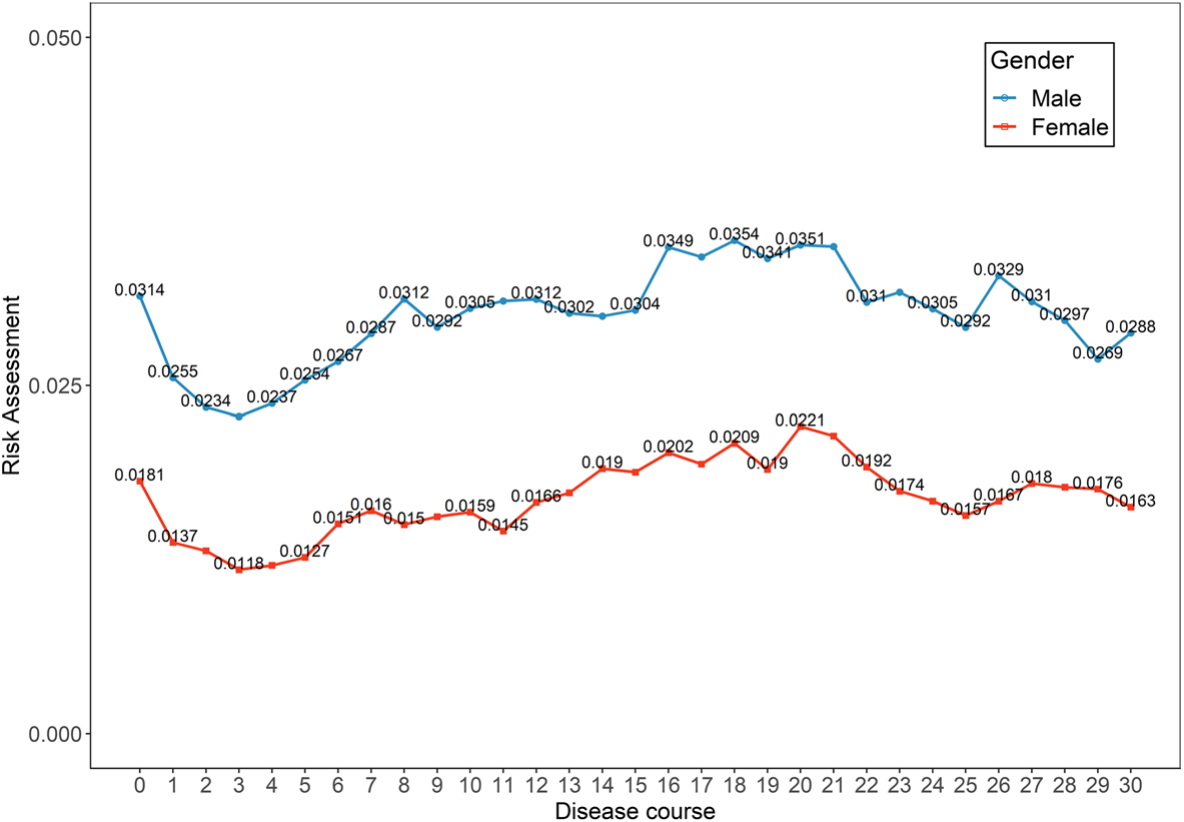
The change of risk assessment with the course of disease by gender with increasing trend (Female, *p* < 0.001)

**Fig. 6:**
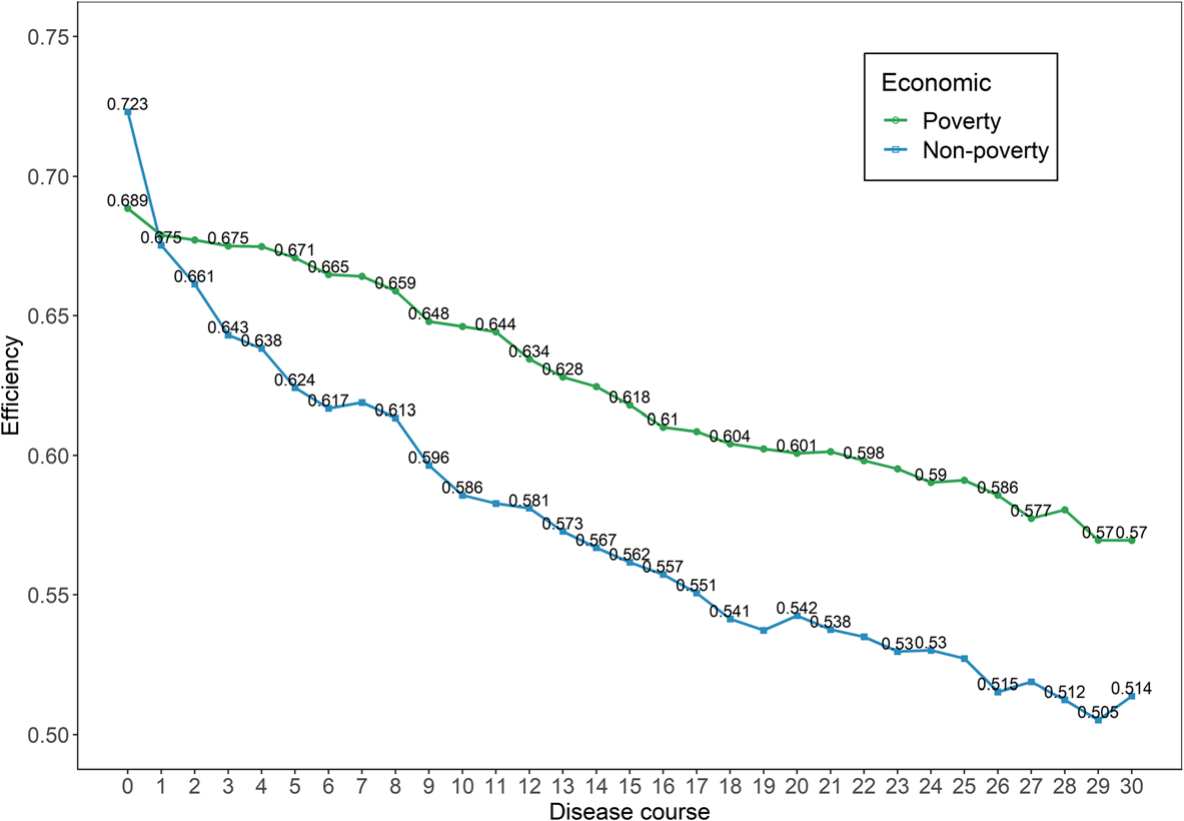
The change of effective rate with the course of disease by economic status with decreasing trend (Poverty, *p* < 0.001; Non-poverty, *p* < 0.001)

**Fig. 7:**
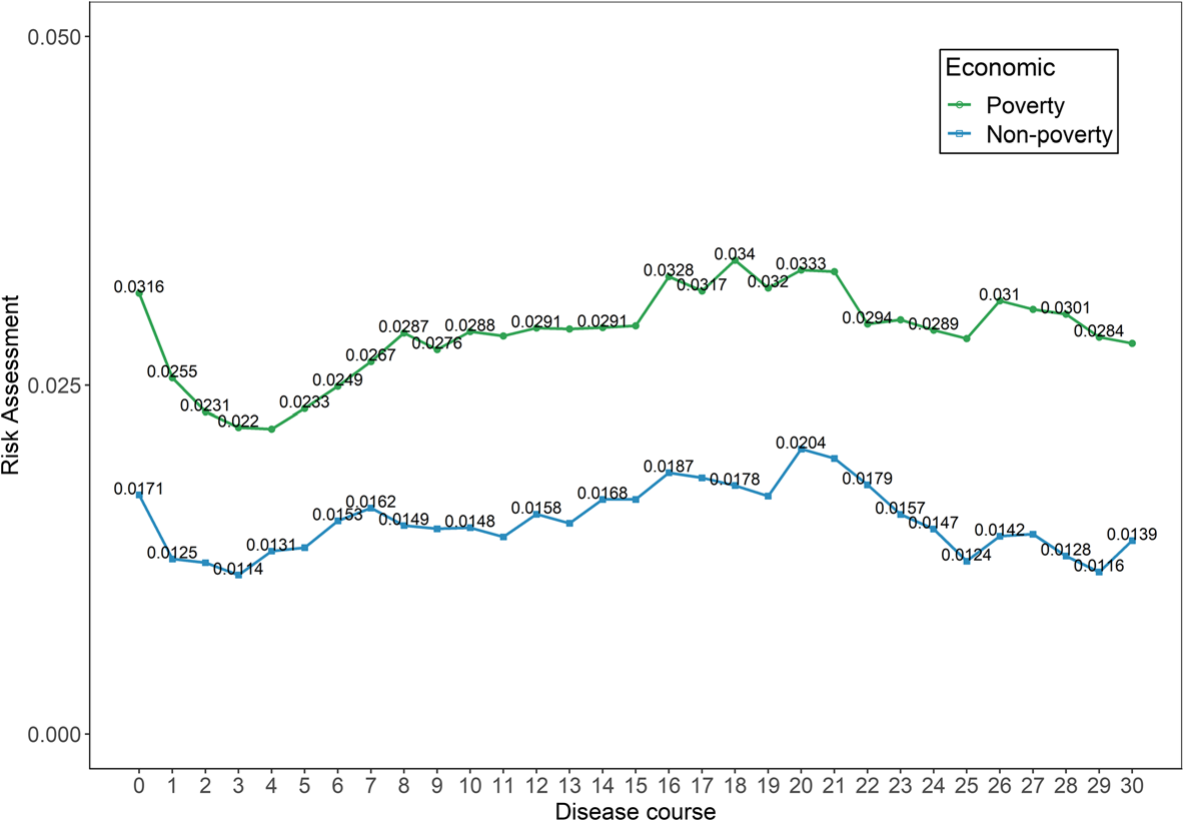
The change of risk assessment with the course of disease by economic status with increasing trend (Poverty, *p* = 0.004)

**Fig. 8:**
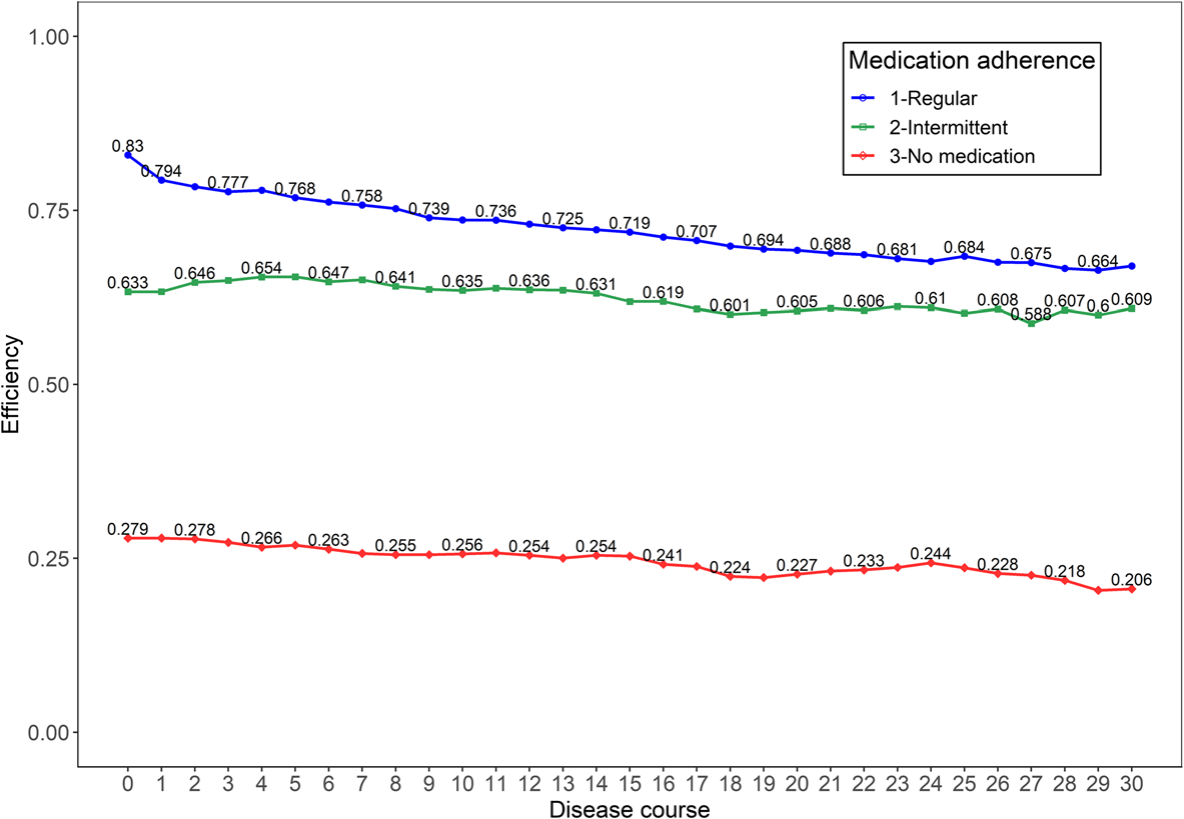
The change of effective rate with the course of disease by medication adherence with decreasing trend (Regular medication, *p* < 0.001; Intermittent medication, *p* < 0.001; No medication, *p* < 0.001)

**Fig. 9:**
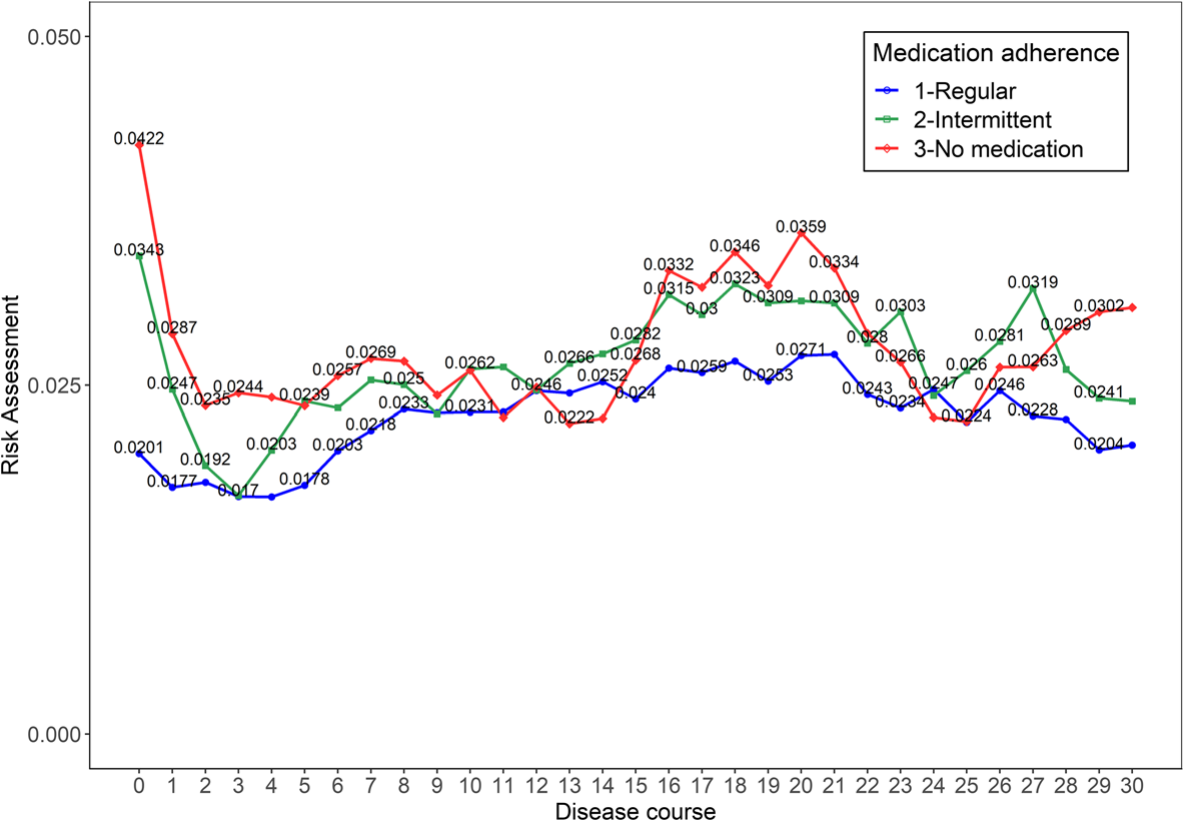
The change of risk assessment with the course of disease by medication adherence

**Fig. 10:**
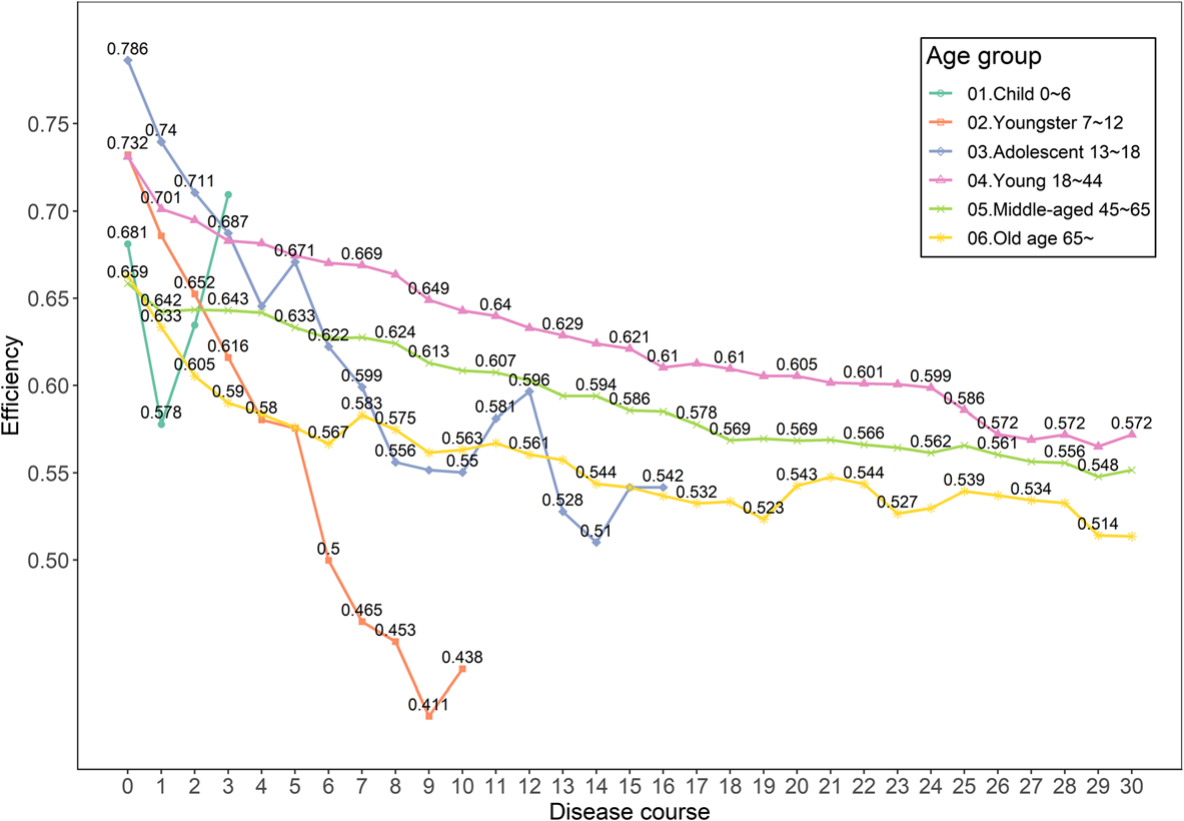
The change of effective rate with the course of disease by age group with decreasing trend (Youngster, *p* = 0.031; Adolescent, *p* = 0.004; Young, *p* < 0.001; Middle-aged, *p* < 0.001; Old age, *p* < 0.001)

**Fig. 11:**
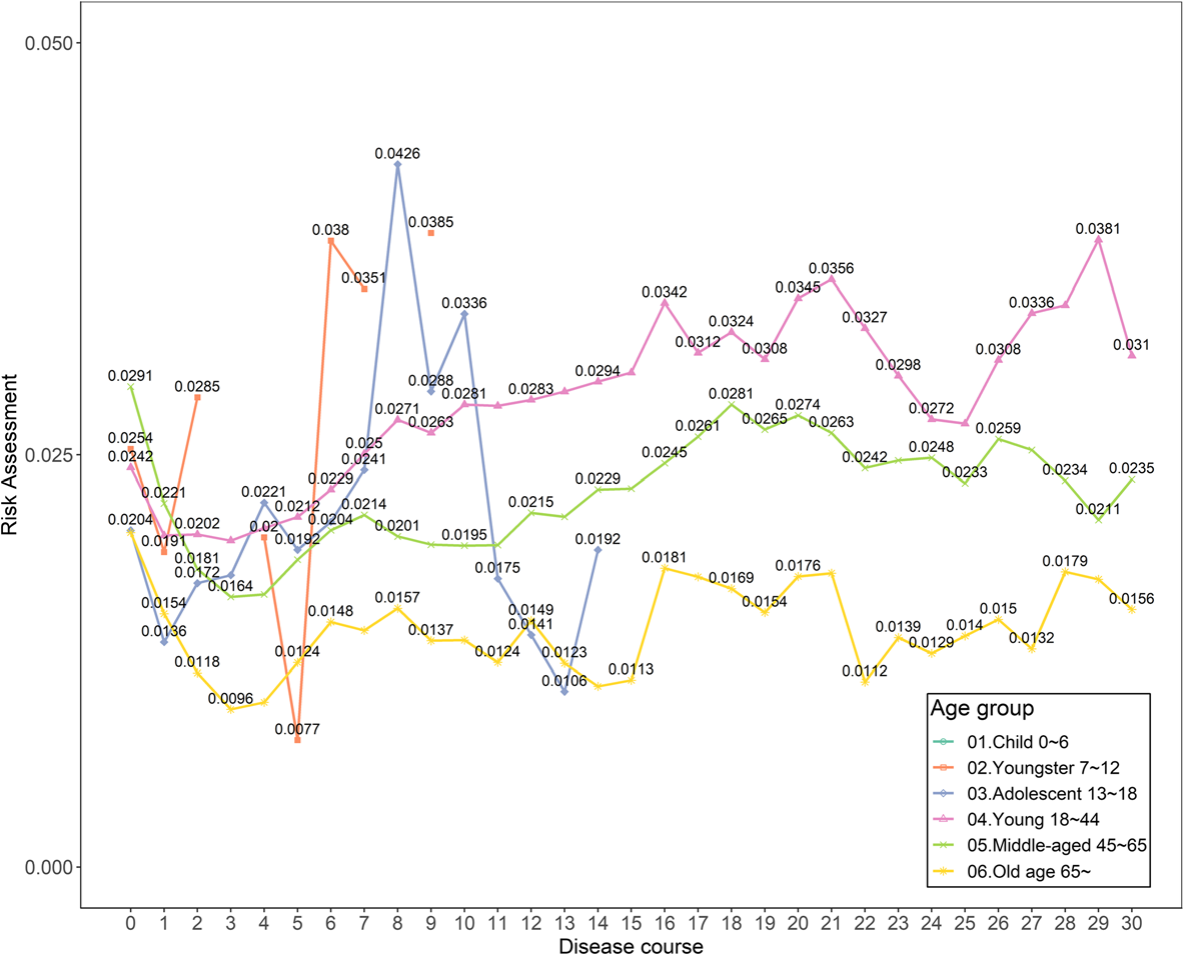
The change of risk assessment with the course of disease by age showing an increasing trend (Young, *p* < 0.001; Middle-aged, *p* = 0.004)

**Fig. 12:**
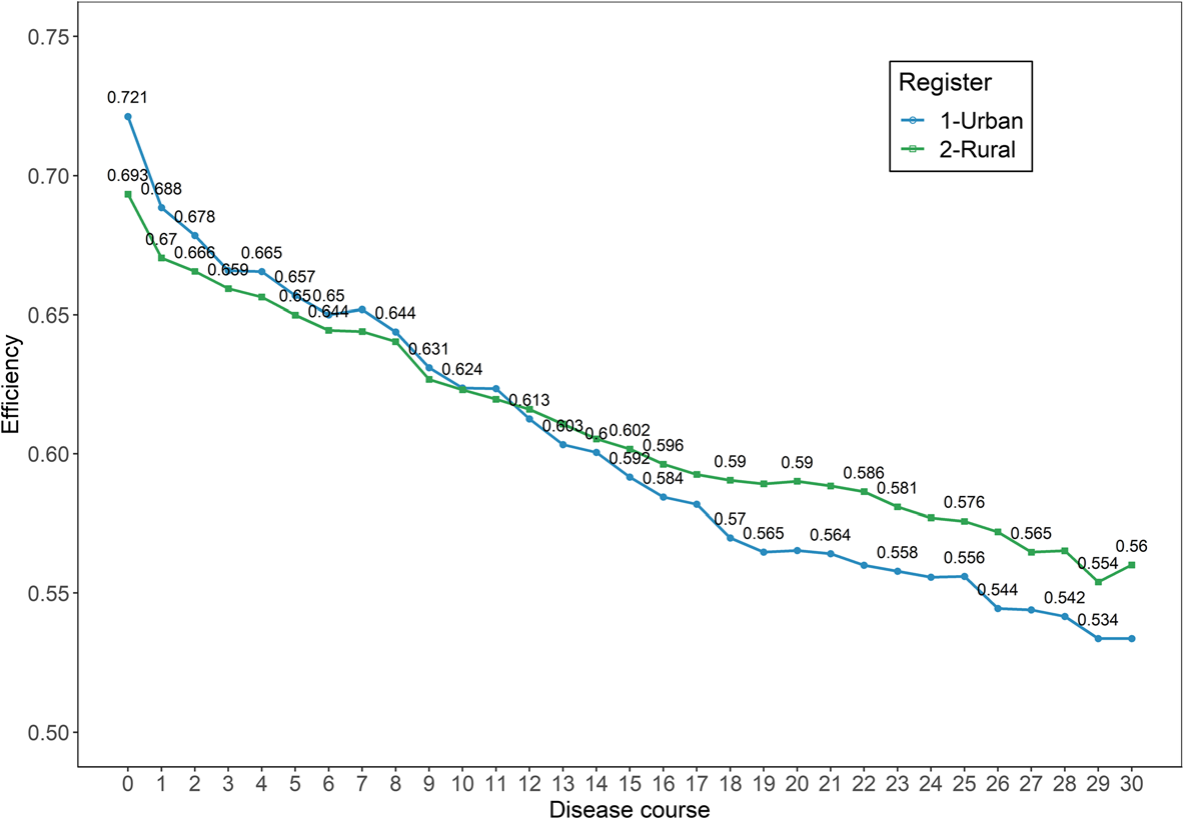
The change of effective rate with the course of disease by registration sites with decreasing trend (Urban area, *p* < 0.001; Rural area, *p* < 0.001)

**Fig. 13:**
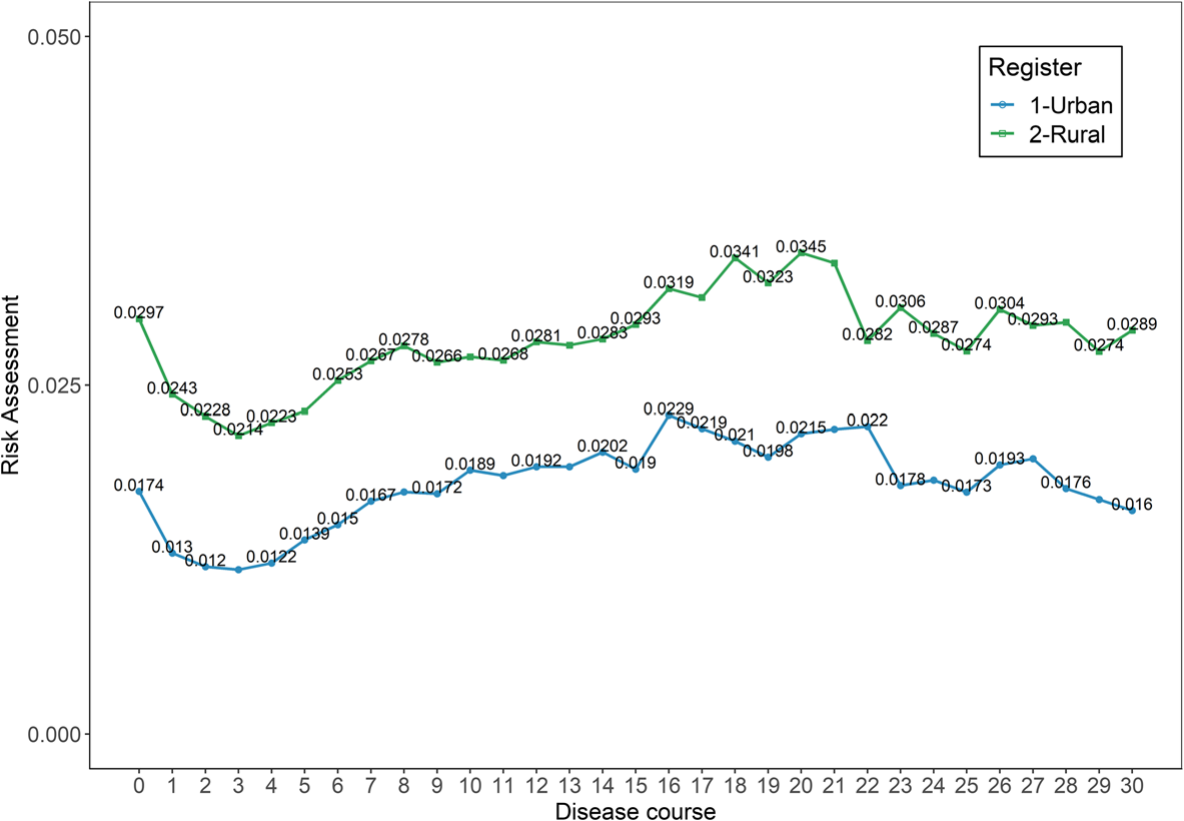
The change of risk assessment with the course of disease by registration sites showing an increasing trend (Urban, *p* = 0.018; Rural, *p* < 0.001)

**Fig. 14:**
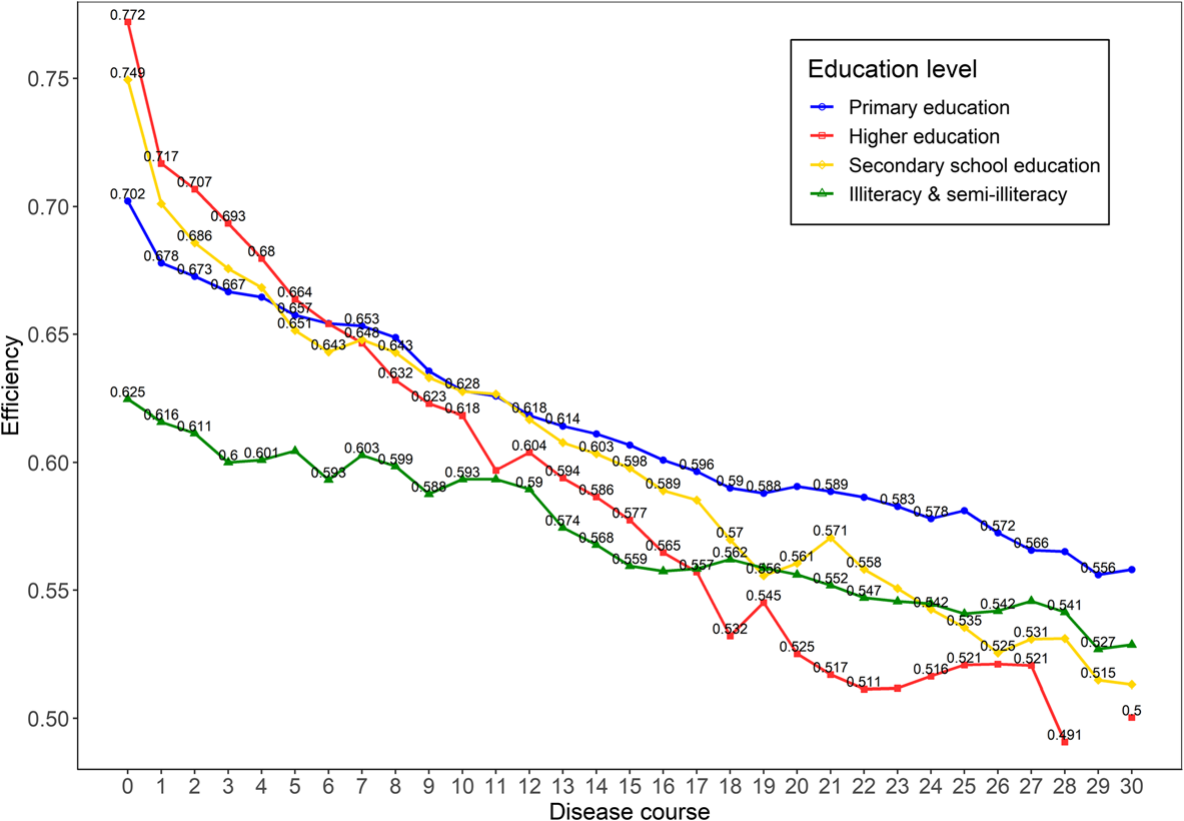
The change of effective rate with the course of disease by education level with decreasing trend (Primary education, *p* < 0.001, Higher education, *p* < 0.001; Secondary school education, *p* < 0.001; Illiteracy&semi-illiteracy, *p* < 0.001)

**Fig. 15:**
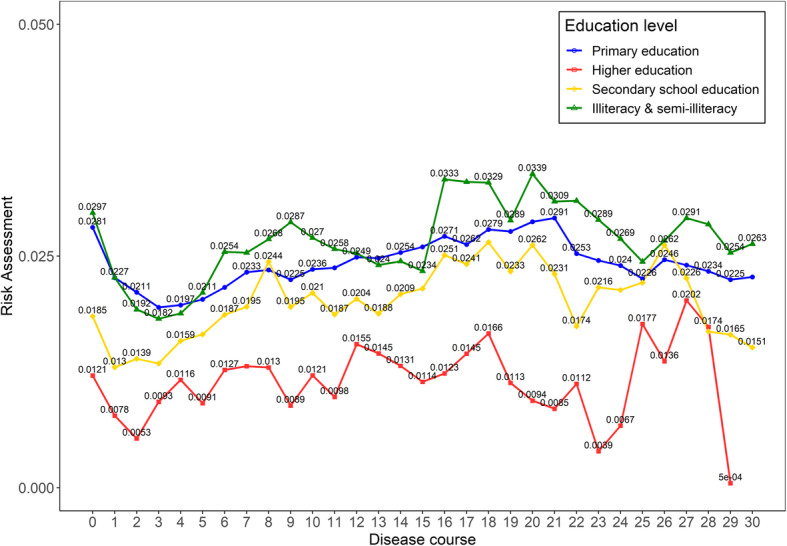
The change of risk assessment with the course of disease by education level showing an increasing trend (Illiteracy&semi-illiteracy, *p* = 0.004)

**Fig. 16:**
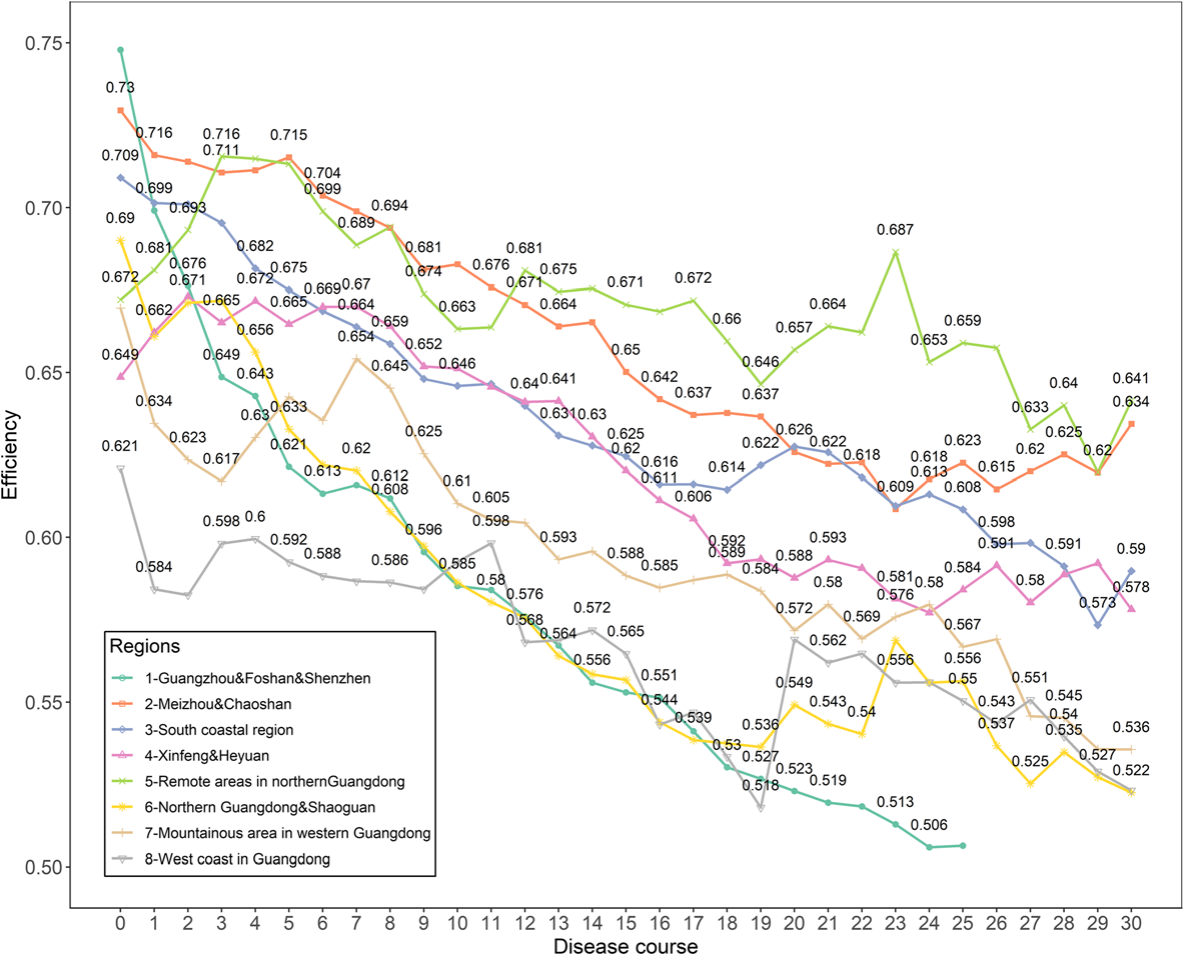
The change of effective rate with the course of disease by major regions in Guangdong province With decreasing trend (Guangzhou&Foshan&Shenzhen, *p* < 0.001; Meizhou&Chaoshan, *p* < 0.001; South coastal region, *p* < 0.001; Xinfeng & Heyuan, *p* < 0.001; Remote areas in northern Guangdong, *p* < 0.001; Northern Guangdong & Shaoguan, *p* < 0.001; Mountainous area in western Guangdong, *p* < 0.001; West coast in Guangdong, *p* < 0.001)

**Fig. 17:**
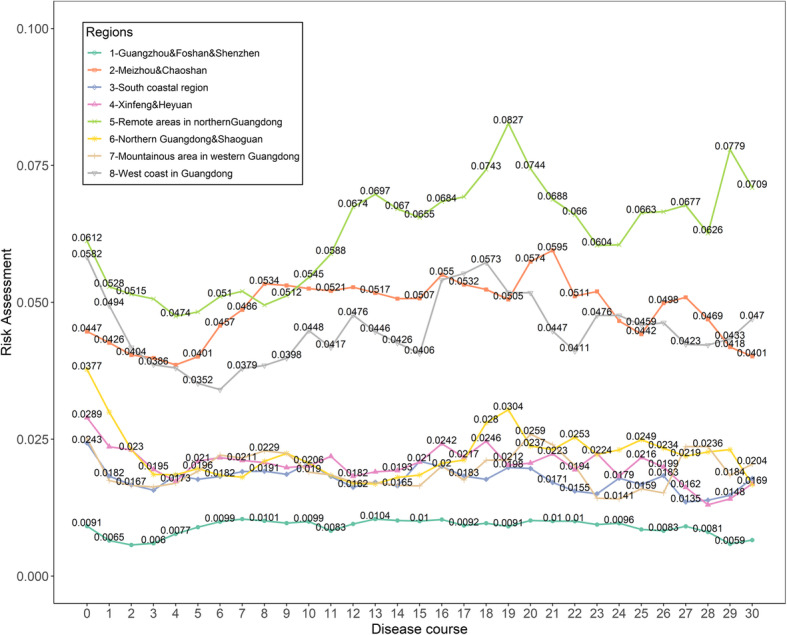
The change of risk assessment with the course of disease by major regions of Guangdong province showing an increasing trend (Remote areas in northern Guangdong, *p* < 0.001; Northern Guangdong & Shaoguan, *p* = 0.018; West coast in Guangdong, *p* = 0.018)

**Fig. 18:**
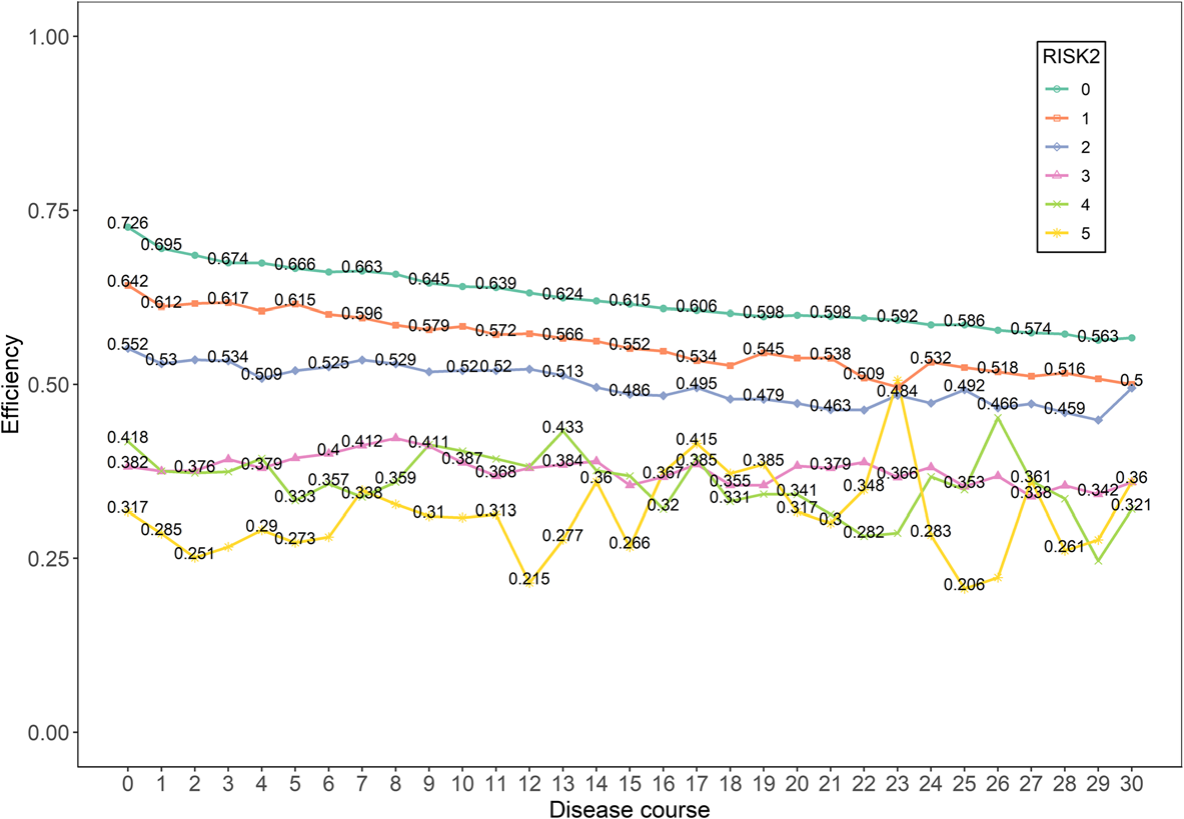
The change of effective rate of with the course of disease by risk levels with decreasing trend (Risk 0, *p* < 0.001; Risk 1, *p* < 0.001; Risk 2, *p* < 0.001; Risk 3, *p* = 0.004; Risk 4, *p* = 0.018)

**Fig. 19:**
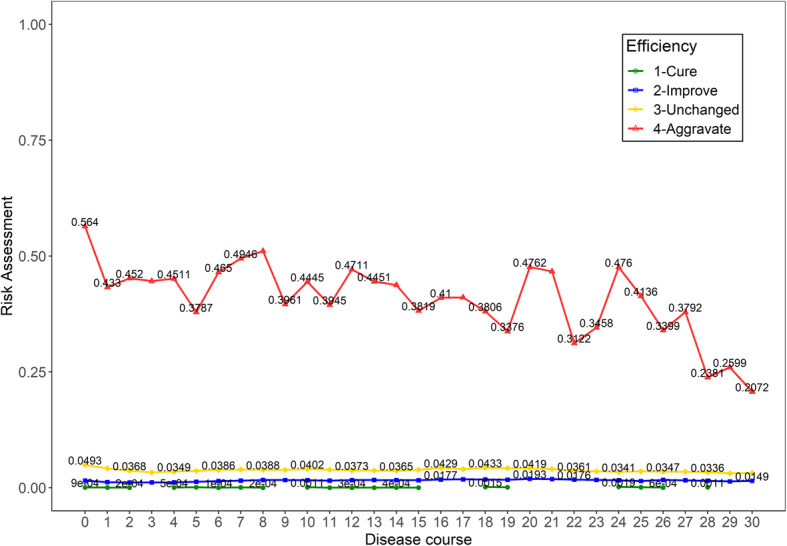
The change of risk assessment with the course of disease by treatment effect showing a decreasing trend (Aggravate, *p* = 0.018)

Overall, the efficacy tended to worse with the course of the disease, and the risk score was higher with the course of the disease. Through statistical analysis of various factors, there was little difference in the effect of gender on efficiency. However, there was a significant difference in the effect of gender on risk score, demonstrating that the risk score of men was higher than that of women (Figs. 4 and 5). The effect of economic conditions for treatment efficacy was not prominent (Fig. 6). When the course of disease exceeded 1 year, the treatment efficiency of poor economic condition was relative higher. While the effect of economic conditions on the risk score was more obvious, reflecting in the fact that poorer economic conditions had higher risk scores at each time point in the disease course compared with better economic conditions, and the risk score existed a certain gap between different economic conditions (Fig. 7). Medication adherence exerted a significant effect on the therapeutic efficiency (Fig. 8). Three different grades of medication adherence indicated that better medication adherence represented higher therapeutic efficiency. In particular, there was a prominent difference between the effective rate of intermittent medication and non-medication, suggesting that drugs played a certain role in the improvement of psychiatric treatment. As for risk assessment, there was little difference between medication compliance levels and a certain degree of fluctuation with the development of disease course. In general, risk assessments for regular medication adherence were lower (Fig. 9). Dividing the age groups by six groups manifested that the effective rate of young people and middle-aged and elderly people was relatively higher, and the effective rate of youngsters shown a relatively rapid decline in the first 10 years of the course of disease, while young people had a higher risk score in the overall course of disease distribution (Figs. 10 and 11). In view of the distribution of efficiency and risk in urban and rural areas, the efficiency of urban environment was higher in the first 10 years of disease course, while the efficiency of rural was overpassed the urban when the disease course continues to increase. In terms of risk, the risk score of urban areas was lower than that of rural areas in each disease course, which existed some certainly consistent with the analysis of the corresponding economic status (Figs. 12 and 13). According to the classification of the education level of the population, there was little difference in the treatment efficiency among different education levels, but the patients who had received higher education tended to have the highest treatment efficiency at the beginning of the disease, while the illiterate and semi-literate patients had a lower treatment efficiency (Fig. 14). In terms of risk assessment, correspondingly, the patients who had received higher education had the lowest risk score in each course of disease, while the risk score of the patients who had been illiterate and semi-literate and primary education was relative higher (Fig. 15). The distribution of treatment efficiency and risk of the major 8 regions of Guangdong province was analyzed and visualized in (Fig. 16**)**. The regions with better economic conditions, such as Guangzhou, Foshan and Shenzhen, had higher treatment efficiency at the initial stage of disease course, but shown the lower treatment effect in the overall course of disease. On the contrary, in the risk assessment, the better economic conditions reflected the lower risk score and the risk was generally higher in the remote areas of north Guangdong (Fig. 17). Correlated the effectiveness and risk, four different treatment effects were classified in the risk assessment according to the patients’ recovery, including cure, improvement, no change and aggravation. It could be seen that the risk assessment value represented by the aggravation of the patient’s illness was the highest and different with the other three indicators. In the analysis of treatment effect, the risk was divided into 6 different grades, and overall the lower risk degree corresponded to the better treatment effect. But there were still some cases where the risk was higher and the treatment effect was also higher. In the risk assessment, because of the incomplete follow-up records of some patients’ cure conditions, data in some disease courses was missed (Figs. 18 and 19).

Furthermore, statistical method (Cox-Stuart) was used to analyze the significance of the risk and therapeutic effect trend in each group. Among them, the intro-group factors whose risk increased significantly with the course of the disease were: urban and rural register factors, illiteracy and semi-illiteracy attributes in education level factors, poverty attributes in economic factors, young people and middle-aged people in age groups, females in gender factors, as well as remote mountainous areas in northern Guangdong in geographical categories. While in the effective rate analysis, the effective rate of each group mostly showed a significant decline with the course of disease, except for the following two groups: level 5 in risk factors and child attributes in age group. These results could also be derived from the corresponding curve trends.

Through the comparison between the different factors within the set of attributes to be efficient and risk difference, we identified that different degrees of medication adherence, different condition of economic, different ages and risk level had difference in treatment effect in general. In the risk assessment, there were significant differences among the attributes of the group in gender, economic status, age group, urban-rural and treatment efficiency.

### Disease distribution analysis

**Fig. 20:**
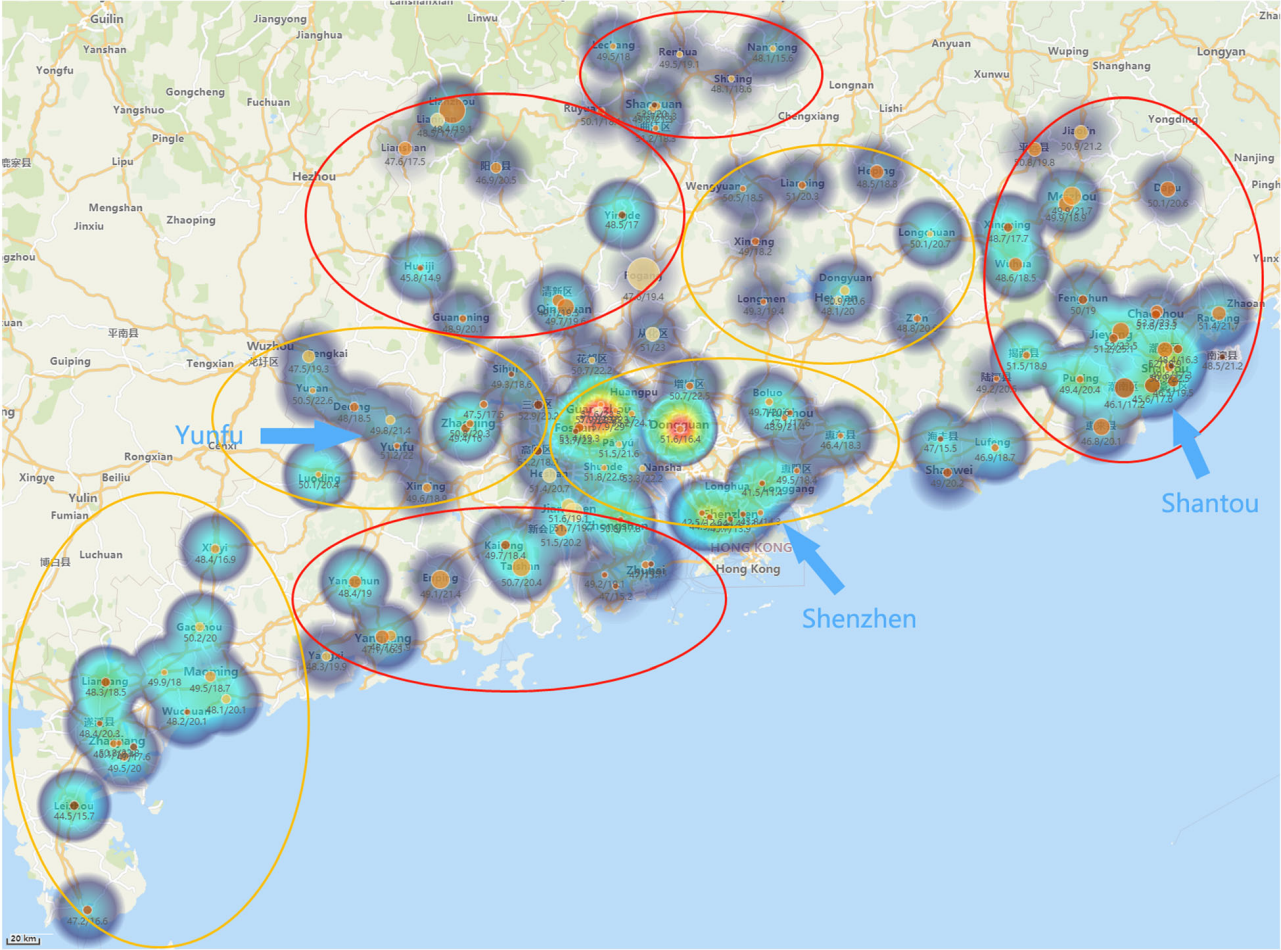
Geographical distribution map of schizophrenia in Guangdong province in terms of treatment efficiency and risk assessment

A hotspot map was used to represent the distribution of effectiveness and risk assessment of the patients, where node colors and node sizes represent the treatment effect and risk assessment respectively, as shown in Fig. 20. By observing the color level mapping to the number of cases, the areas with a large number of cases tended to be distributed in areas with high population density, such as Guangzhou, Dongguan and Chaoshan area. By analyzing the mapping of node colors and sizes, it could be found that in the economically developed cities, such as Foshan, Guangzhou and the Pearl River delta region. The treatment effect was moderate but the risk assessment was generally low. Meanwhile, it could be seen that the treatment effect of schizophrenia was poor and the risk assessment was significantly higher in the remote mountainous area of the rural-urban junction area in north Guangdong, which is relatively backward in economic development, especially in Lianshan county and Fogang county. It worth to be noted that Chaoshan area (including Chaozhou and Shantou cities) was a special area with a large number of patients and a relatively long course of disease. Although this area possessed a certain degree of therapeutic effect, the risk assessment was generally high and the distribution was relatively dense, indicating that this might be an area that needed to be paid more attention to. In southern Guangdong province, some coastal cities, such as Kaiping and Yangjiang, had low population density, but poor treatment effect and high-risk assessment, indicating that these were also one of the regions with serious mental illness in current situation. While in southern coastal areas such as Maoming and Zhanjiang, antipsychotic treatment was more effective and less risky.

Through the above analysis of the distribution characteristics of schizophrenia in various regions of Guangdong province, it could be found that the efficacy and risk were correlated. Poverty areas with backward economic conditions such as northern Guangdong were often accompanied by poor curative effect and high-risk assessment. On the contrary, in areas with developed economy and good medical resources, such as Guangzhou, Foshan, Shenzhen, the risk assessment was lower, indicating that the disease was effectively controlled. Meanwhile, Chaoshan area deserved more attention for its special geographical location and disease distribution condition.

## Discussion

Schizophrenia is a complex mental illness which brings great economic loss to patients and society [25]. This is the first work to carry out epidemiology study of schizophrenia on large quantity of patients with mental illness using follow-up data and to conduct integrated processing and data analysis using a platform of big data analysis. We used statistical analysis method to study the characteristics and impact factors of schizophrenia.

Through this study, the mental health center established a refined treatment effect and disease risk control system based on the quantification of disease risk indicators based on the big data. The system dynamically presented the history and current situation of treatment in each region of Guangdong province through the analysis of patient demographic characteristics, disease conditions, treatment, region and other dimensions. The system could help managers to intuitively compare the prevention and control of schizophrenia as well as the effectiveness of regional health policies. It also enabled managers to better combine data phenomena, regional characteristics and corresponding measures for analysis, continuous attention and in-depth research. For example: 1) Shenzhen was characterized by large population mobility and difficult follow-up of urban patients. By improving service, Shenzhen had carried out joint follow-up visits for patients by mental health social workers and primary schizophrenia prevention doctors, so as to improve follow-up compliance of patients and reduced the occurrence of dangerous behaviors of patients who had been followed up. 2) Every primary health and medical institution in Yunfu city had opened a psychiatric output clinic with the characteristic of complete inpatient and outpatient medical insurance, thus improving the treatment rate and medication compliance of patients. 3) In response to the poor medical conditions in rural areas, Shantou city had established a “mental health prevention day”, in which psychiatrists from municipal psychiatric hospitals regularly send medicine to the countryside free of charge. To some extent, this alleviated the difficulty in seeking medical treatment and the inconvenience in the return visit, so that patients could return visit and take medicine in a timely manner. The system would also continue to improve in data governance, quality control, analysis and mining functions, and would provide more help for the study of regional differences, measures, and effectiveness of the causal relationship.

In the study, we conducted clinical staging of the patients’ course to analyze the characteristics of the disease and treatment in different populations and stages of real-world schizophrenia. Clinical staging, as an important basis of clinical treatment, had been widely used in the diagnosis and treatment of various diseases. Moreover, clinical staging was of great significance for the improvement of clinical treatment outcome. In the field of schizophrenia, the Royal Australian and New Zealand College of Psychiatrists had published the guidelines for clinical practice management of schizophrenia and related disorders in 2016, which proposed a new concept of schizophrenia staging and a corresponding strategy of step-based treatment [26]. Therefore, studying the disease characteristics of schizophrenia in different stages and carrying out corresponding preventive intervention was of great meaningful, which could effectively avoid the transformation of the disease. Our results demonstrated the distribution characteristics of disease and treatments at different stages of disease course, which provided references for personalized treatment, optimization of disease prevention and control.

Statistics on the age of initial onset by males and females were conducted respectively. Although recent studies suggest that differences in prevalence between male and female were controversial [27], the results manifested that these differences might be existed in current regional characteristics. By observing that the number of male patients was higher than female before the age of 30 to 34 years old but fewer than female after 34 years old, we believed that the social factors and physiological characteristics contributed to gender differences in schizophrenia, and might also have a significant impact on the prevalence of psychosis.

In our study, several designed factors were selected, which might be relevant to the efficiency and risk analysis of schizophrenia. By comparing the differences in efficacy and risk among intra-group attributes of different factors, our results revealed that treatment efficacy presented differences among different degrees of medication adherence, different condition of economic, different age groups and risk levels. As suggested in [28, 29], patients with mental illness who persist in antipsychotic treatment without interruption of medication could effectively improve the therapeutic effect of the disease. Thus, we possibly needed to pay more attention to these factors that were sensitive to schizophrenia and had a potential impact on the improvement of treatment outcome. In the risk analysis, there were certain differences among the attributes among groups by gender, economic status, age group, urban-rural and treatment efficiency. The gender difference also reflected our analysis results of the number of cases in male and female patients with different disease course, showing that male had a higher risk of disease than female. Economic conditions partly reflected the relationship between schizophrenia and treatment conditions. The treatment of psychosis tended to bring the higher costs [30]. Families with better economic conditions probably were more willing to accept long-term treatment for schizophrenia, while families with poor economic conditions might abandon treatment because of the high cost, leading to further deterioration of the disease. These differences and trends indicated that certain factors and environment were associated with the development of psychosis, which had guiding significance for the treatment of schizophrenia.

We took the location factor into account and converted the efficacy into efficient density layer, which was supposed to be better demonstrate the efficacy distribution. Our results suggested that social factors might influence the outcome and risk of rural urban gradients of psychosis. This was meaningful since it implied that some social determinants of the geographical distribution of psychosis-including socioeconomic or population size were influencing factors. The geographical distribution of schizophrenia could be explained as follows. In economically developed regions, the medical conditions and awareness of patients with schizophrenia were relatively perfect, and patients had more opportunities to receive professional antipsychotic treatments, so that the disease could be better managed and the risk assessment was relatively lower. On the contrary, in the places with relatively backward economy and large population, lower education attainment, backward medical conditions, lower economic status, less access to health services, and lover income contributed to the possibility that patients could not afford the high treatment costs or missed the best treatment time due to less awareness of schizophrenia [31, 32]. The socioeconomic and population sizes had been suggested that risk factors for schizophrenia were cumulative and interactive, both with each other [33]. The geographical nature of disease distribution also suggested that we needed a major redistribution of social and health resources to address this inequities issue, and this happened if strong political, economic, social and professional stakeholders in the community were actively involved.

Because of the large population of China, a small prevalence rate could lead to a large number of cases. As mental disorders usually bring substantial disease burden, the Government needed pay more attention to mental health care [34]. With respect to this, there were initiatives to establish universal health care coverage, and a growing number of individuals were covered by health insurance systems, which could provide some relief from the high direct health care costs of schizophrenia for the patient and family members [35, 36]. Because of common mental disorders were associated with greater impairment than chronic physical disorders but were markedly undertreated, it is indicating that the priority of China’s medical resources allocation should be actively adjusted, and the use of medical resources should be optimized [37]. For the treatment of schizophrenia in Guangdong province, we should pay more attention to some relatively poor areas, such as the remote mountainous areas in north and west Guangdong, to achieve a reasonable allocation of medical resources. Moreover, Chaoshan area with its high risk and complex disease characteristics is another area worthy of attention.

A long-time follow-up was conducted in this study, and the recorded data lasted for 45 years, which was a relatively large and important workload. A large number of follow-up data were collected during the long-term disease registration and follow-up management, including 20 million follow-up data in the last decade. We recorded the data in detail, including: 1) demographic sociology data; 2) psychiatry diagnosis, symptoms, treatment and rehabilitation measures, risk, social function assessment, etc.; 3) medication status and adverse reactions of patients; and 4) a series of other information. The abundant data was conducive to the epidemiological study of schizophrenia and the analysis of the role of several factors, including some demographic factors and environmental geographical distribution. Here, we designed the correlated formula to quantify the therapeutic effect and risk to better assess the impact of different factors on psychosis. The factors with significant differences among different attributes in the group were focused to provide reference basis for evaluating and optimizing the treatment and follow-up management of mental disease as well as accurate disease prevention and control. Elaborately depicting the regional distribution characteristics of disease, exploring the differences of therapeutic effect and risk in different regions, and proposing the regions that need to be paid more attention to, which provides help for the corresponding policy making and health institutions to provide more targeted services. In the future, we will continue to conduct in-depth research to explore how to better integrate our research results with relevant clinical work, so as to provide references for clinical personalized treatment, optimization of disease prevention and control and other work.

## Conclusions

The evidence of variation in therapeutic effect and risk assessment in different regions, associated with local economic levels and medical resources was analyzed. Several demographic factors affecting the effectiveness and risk of psychiatric care were identified, which might benefit the evaluation and optimization of the disease treatment. Through the analysis, a list of high-risk areas in Guangdong province should be paid more attention to achieve effective and reasonable allocation of medical resources for improving the overall treatment status of schizophrenia in the province.

## Data Availability

Data are provided by Guangdong Mental Health center and it cannot be shared with other research groups without necessary permission.

## Abbreviations

CMHS: The China Mental Health Survey
ICD: International Classification of Disease
GDMHS: Guangdong Mental Health center network medical System

## References

[1] Naber D, Hansen K (2015). Qualify: a randomized head-to-head study of aripiprazole once-monthly and paliperidone palmitate in the treatment of schizophrenia. Schizophr Res.

[2] Brown S, Kim M, Mitchell C, Inskip H (2010). Twenty-five year mortality of a community cohort with schizophrenia. Br J Psychiatry.

[3] Kessler RC (2007). Age of onset of mental disorders: a review of recent literature. Curr Opin Psychiatry.

[4] Charlson FJ, Baxter AJ (2015). Excess mortality from mental, neurological and substance use disorders in the global burden of disease study 2010. Epidemiol Psychiatric Sci.

[5] Saha S, Chant D, McGrath J (2007). A systematic review of mortality in schizophrenia: is the differential mortality gap worsening over time?. Arch Gen Psychiatry.

[6] Janoutová J, Janáčková P. et al, Epidemiology and risk factors of schizophrenia. 2016;37(1):1–8. 26994378

[7] McGrath JJ. The Surprisingly Rich Contours of Schizophrenia Epidemiology. 2007;64:14–6. 10.1001/archpsyc.64.1.14 17199050

[8] MacMahon B, Pugh TF (1970). Epidemiology: principles and methods.

[9] McGrath JJ, Mortensen PB, Whiteford HA (2018). Pragmatic psychiatric epidemiology-if you Can’t count it, It Won’t Count. JAMA Psychiatry.

[10] Ran MS, Yang LH (2017). The family economic status and outcome of people with schizophrenia in Xinjin, Chengdu, China: 14-year follow-up study. Int J Soc Psychiatry.

[11] Jongsma HE, Turner G, Kirkbride JB, Jones PB (2019). International incidence of psychotic disorders, 2002–17: a systematic review and meta-analysis. Lancet Public Health.

[12] Aleman A, Kahn RS, Selten JP (2003). Sex differences in the risk of schizophrenia: evidence from meta-analysis. Arch Gen Psychiatry.

[13] Kirkbride JB, Hameed Y (2017). The epidemiology of first-episode psychosis in early intervention in psychosis services: findings from the social epidemiology of psychoses in East Anglia [SEPEA] study. Am J Psychiatr.

[14] Richardson L, Hameed Y (2018). Association of Environment with the risk of developing psychotic disorders in rural populations findings from the social epidemiology of psychoses in East Anglia study. JAMA Psychiatry.

[15] McGrath JJ, Susser ES (2009). New directions in the epidemiology of schizophrenia. Med J Aust.

[16] Montgomery W, Liu L (2013). The personal, societal, and economic burden of schizophrenia in the People’s republic of China: implications for antipsychotic therapy. Clinicoecon Outcomes Res.

[17] Charlson FJ, Ferrari AJ (2018). Global epidemiology and burden of schizophrenia: findings from the global burden of disease study 2016. Schizophr Bull.

[18] Chan KY, Zhao FF (2015). Prevalence of schizophrenia in China between 1990 and 2010. J Glob Health.

[19] Li XH, Song JC (2016). Urbanization and health in China, thinking at the national, local and individual levels. Environ Health.

[20] Huang YQ, Liu ZR (2016). The China mental health survey (CMHS): I. background, aims and measures. Soc Psychiatry Psychiatr Epidemiol.

[21] Liu ZR, Huang YQ (2016). The China mental health survey: II. Design and field procedures. Soc Psychiatry Psychiatr Epidemiol.

[22] Long J, Huang G (2014). The prevalence of schizophrenia in mainland China: evidence from epidemiological surveys. Acta Psychiatr Scand.

[23] Phillips MR, Zhang JX (2009). Prevalence, treatment, and associated disability of mental disorders in four provinces in China during 2001–05: an epidemiological survey. Lancet.

[24] Shen YC, Zhang MY, et al. Twelve-month prevalence, severity, and unmet need for treatment of mental disorders in metropolitan China. Psychol Med. 2006:257–67. 10.1017/S0033291705006367 16332281

[25] Hu TW (2006). Perspectives: an international review of the national cost estimates of mental illness, 1990–2003. J Ment Health Policy Econ.

[26] Galletly C, Castle D (2016). Royal Australian and new Zealand College of Psychiatrists clinical practice guidelines for the management of schizophrenia and related disorders. Aust N Z J Psychiatry.

[27] Ochoa S, Usall J, Cobo J, et al. Gender differences in schizophrenia and first-episode psychosis: a comprehensive literature review. Schizophr Res Treat. 2012;576369. 10.1155/2012/916198 PMC3420456 22966451

[28] Higashi K, Medic G (2013). Medication adherence in schizophrenia: factors influencing adherence and consequences of nonadherence, a systematic literature review. Ther Adv Psychopharmacol.

[29] Perkins DO (2002). Predictors of noncompliance in patients with schizophrenia. J Clin Psychiatry.

[30] Zhai J, Guo XF (2013). An investigation of economic costs of schizophrenia in two areas of China. Int J Ment Heal Syst.

[31] Byrne M, Agerbo E (2004). Parental socio-economic status and risk of first admission with schizophrenia. Soc Psychiatry Psychiatr Epidemiol.

[32] Scior K, Potts HW (2013). Awareness of schizophrenia and intellectual disability and stigma across ethnic groups in the UK. Psychiatry Res.

[33] Davis J, Eyre H (2016). A review of vulnerability and risks for schizophrenia: beyond the two hit hypothesis. Neurosci Biobehav Rev.

[34] Huang YP, Wang Y (2019). Prevalence of mental disorders in China: a cross-sectional epidemiological study. Lancet Psychiatry.

[35] Liu J, Ma H (2011). Mental health system in China: history, recent service reform and future challenges. World Psychiatry.

[36] Dong KY (2009). Medical insurance system evolution in China. China Econ Rev.

[37] Lee S, Guo WJ (2009). Impaired role functioning and treatment rates for mental disorders and chronic physical disorders in metropolitan China. Psychosom Med.

